# Analysis of serum macrophage migration inhibitory factor and D‐dopachrome tautomerase in systemic sclerosis

**DOI:** 10.1002/cti2.1042

**Published:** 2018-12-06

**Authors:** Fabien B Vincent, Emily Lin, Joanne Sahhar, Gene‐Siew Ngian, Rangi Kandane‐Rathnayake, Rachel Mende, Alberta Y Hoi, Eric F Morand, Tali Lang, James Harris

**Affiliations:** ^1^ Centre for Inflammatory Diseases School of Clinical Sciences at Monash Health Monash University Clayton VIC Australia; ^2^ Department of Rheumatology Monash Health Clayton VIC Australia; ^3^ Present address: The Szalmuk Family Department of Medical Oncology Cabrini Institute Malvern VIC 3144 Australia

**Keywords:** biomarker, cytokine, systemic lupus erythematosus (SLE), systemic sclerosis

## Abstract

**Objectives:**

Macrophage migration inhibitory factor (MIF) and D‐dopachrome tautomerase (DDT), members of the same cytokine superfamily, are linked to the pathogenesis of a number of inflammatory diseases. The aim of this study was to investigate their clinical relevance in systemic sclerosis (SSc).

**Methods:**

Serum MIF and DDT were quantified in 105 SSc patients by ELISA and levels compared to healthy controls (HC) (47) and patients with systemic lupus erythematosus (SLE) (184). Clinical parameters included organ involvement, serum laboratory markers and results of pulmonary function tests, and overall disease activity assessed using the European Scleroderma Trials and Research group (EUSTAR) activity index.

**Results:**

There was no significant difference in serum DDT concentrations between patients with SSc and HC. However, serum MIF was significantly increased in SSc compared to both HC and SLE cohorts. Serum MIF was increased in SSc patients with low forced vital capacity (FVC) and was also associated with the use of angiotensin II receptor blockers and beta blockers in SSc, confirmed after adjusting for the presence of systemic hypertension and low FVC. Serum DDT was significantly higher in SSc patients with low FEV1 and negatively correlated with EUSTAR score, particularly in patients with limited disease.

**Conclusion:**

Although not significantly linked to specific clinical parameters, serum MIF was significantly higher in SSc patients than in HC and SLE patients, suggesting a fundamental role for MIF in SSc. DDT, while closely related to MIF, did not show a similar expression profile, suggesting functional differences between these molecules.

## Introduction

Systemic sclerosis (SSc, scleroderma) is a chronic, multisystem autoimmune disease characterised by fibrosis, vascular dysfunction and immune dysregulation, notable for its biological and clinical heterogeneity.^1^, ^2^ While genetic and environmental factors are implicated in SSc disease progression, there is also increasing evidence to support a role for dysregulation of the innate immune system in SSc pathogenesis.^1^, ^2^

Macrophage migration inhibitory factor (MIF) is a pleiotropic inflammatory molecule with a broad range of immunomodulatory properties.^3^ MIF has been shown to play a role in disease progression of autoimmune and inflammatory disorders, including rheumatoid arthritis, systemic lupus erythematosus (SLE), inflammatory bowel disease and multiple sclerosis.^3^, ^4^, ^5^ Studies have suggested possible associations between MIF and SSc, but the relationship between the two is unclear. In particular, MIF polymorphisms associated with increased serum MIF levels have been linked to SSc disease severity in European and North American populations,^6^, ^7^, ^8^ while small cohort studies have linked high levels of serum and tissue MIF with pulmonary arterial hypertension (PAH) and digital ulcers in diffuse SSc.^9^, ^10^ A second member of the MIF superfamily, D‐dopachrome tautomerase (DDT; MIF‐2), has also been identified, with similar physiological and biochemical properties to MIF.^11^ In particular, DDT is found in most tissues and is present at similar levels to MIF in serum and the two proteins appear to work cooperatively.^12^ DDT has been associated with pathology in a number of diseases, including multiple sclerosis.^4^ To date, DDT has not been investigated in SSc.

Here, we looked for clinical associations of serum MIF and DDT in a well‐characterised SSc cohort. In addition, we compared serum MIF levels of SSc patients to SLE patients, as an autoimmune disease control group, and healthy controls.

## Results

### Participant characteristics

A total of 105 SSc patients were included in this study (Table 1). Mean (SD) age and median [IQR] disease duration were 60.1 (13.9) and 12.3 [6.8, 19.3] years, respectively. Patients were predominantly female (82.9%) and Caucasian (83.5%). Twenty‐two per cent of patients had diffuse disease, and the median [IQR] modified Rodnan skin score (MRSS) was 5 [3, 8]. Median [IQR] European Scleroderma Trials and Research group score was 1.5 [0.5, 2.5], with 27% of patients classified as having active disease. SLE and healthy controls (HC) cohorts consisted of 184 patients and 47 individuals, respectively. There was a statistically significant difference in age, gender and ethnicity between the groups (Supplementary table 1); these were adjusted for in statistical analyses.

**Table 1:** SSc patient demographics and disease characteristics

	SSc patients (*n* = 105)
Demographics	
Age (years), mean (SD)	60.1 (13.9)
Female, *n* (%)	87 (82.9%)
Ethnicity*, *n* (%)	
Caucasian	86 (83.5%)
Asian	10 (9.7%)
Other	7 (6.8%)
Clinical details	
Disease duration (years), median [IQR] (range)	12.3 [6.8, 19.3] (0.6, 46.7)
Diffuse SSc, *n* (%)	23 (21.9%)
EUSTAR**, median [IQR] (range)	1.5 [0.5, 2.5] (0, 5)
Patients with active disease (EUSTAR ≥2.5), *n* (%)	22 (27%)
Clinical manifestation	
Pulmonary arterial hypertension, *n* (%)	5 (4.8%)
Pericardial effusion, *n* (%)	5 (4.8%)
Interstitial lung fibrosis, *n* (%)	35 (33.3%)
Systemic hypertension*, *n* (%)	32 (32%)
Renal crisis, *n* (%)	4 (3.8%)
Digital ulcers*, *n* (%)	14 (14%)
mRSS*, median [IQR] (range)	5 [3, 8] (0, 20)
mRSS >18, *n* (%)	1 (1%)
Gastrointestinal^a^, *n* (%)	61 (58.1%)
GAVE, *n* (%)	9 (8.6%)
Reflux oesophagitis, *n* (%)	59 (56.2%)
Oesophageal stricture, *n* (%)	9 (8.6%)
Oesophageal dysmotility, *n* (%)	5 (4.8%)
Bowel dysmotility, *n* (%)	2 (1.9%)
Raynaud's phenomenon*, *n* (%)	85 (85%)
Calcinosis*, *n* (%)	23 (23%)
Myositis, *n* (%)	2 (1.9%)
Synovitis*, *n* (%)	11 (11%)
Pulmonary and cardiac function tests	
FVC (%)*, mean (SD)	93.7 (18.2)
FEV1 (%)*, mean (SD)	89.7 (18.3)
DLCO (%) b,***, median [IQR] (range)	59.5 [48.1, 73.6] (24.6, 116.4)
KCO (%) c, ****, mean (SD)	64.4 (17.2)
Six‐minute walk distance (m)*****, median [IQR] (range)	508 [432, 560] (252, 697)
LVEF (%)******, median [IQR] (range)	65 [60, 65] (35, 75)
sPAP (mmHg) ******, median [IQR] (range)	31 [28, 39] (21, 108)
Clinical laboratory data	
ANA +ve*, *n* (%)	100 (96.2%)
ANA anti‐centromere +ve*, *n* (%)	42 (40.4%)
Anti‐topoisomerase I*, *n* (%)	25 (24.3%)
Anti‐RNA polymerase III +ve*, *n* (%)	9 (8.8%)
CRP (mg/L) ***, median [IQR] (range)	3.5 [1.4, 6] (0.2, 46)
ESR (mm/h) *******, median [IQR] (range)	10 [5, 17] (1, 77)
Creatinine (μmol/L) ********, median [IQR] (range)	65 [54, 76] (36, 149)
Treatment, *n* (%)	
Glucocorticoids	24 (22.9%)
Hydroxychloroquine	14 (13.3%)
Immunosuppressants^d^	23 (21.9%)
Biologics^e^	1 (1%)
PDE5 inhibitor	5 (4.8%)
ERA	5 (4.8%)
Ca2^+^ channel antagonist	51 (48.6%)
Anticoagulant	7 (6.7%)
Anti‐platelet agent	19 (18.1%)
ACE inhibitor	11 (10.5%)
Angiotensin II receptor blockers	17 (16.2%)
Beta blockers	5 (4.8%)

ANA, antinuclear antibodies; CRP, C‐reactive protein; DLCO, Hb‐ and gender‐ corrected diffusing capacity of the lungs for carbon monoxide; ERA, endothelin receptor antagonist; ESR, erythrocyte sedimentation rate; EUSTAR, European Scleroderma Trials and Research; FEV1, forced expiratory volume in one second; FVC, forced vital capacity; GAVE, gastric antral vascular ectasia; LVEF, left ventricular ejection fraction; MIF, macrophage migration inhibitory factor; mRSS, modified Rodnan skin score; PDE5, phosphodiesterase 5; Sm, Smith; sPAP, systolic pulmonary arterial pressure; SSc, systemic sclerosis. *≤5 missing values; ** 23 missing values; *** 10 missing values; **** 7 missing values; ***** 76 missing values; ****** 28 missing values; ******* 12 missing values; ******** 8 missing values.

a Includes GAVE, reflux oesophagitis, oesophageal stricture, oesophageal dysmotility, bowel dysmotility and episodes of pseudo obstruction.

b Corrected for haemoglobin and gender.

c DLCO corrected for lung volume.

d Includes leflunomide, methotrexate, azathioprine, mycophenolate, cyclophosphamide and calcineurin inhibitors.

e Anti‐CD20 antibody.

### Serum MIF and DDT in SSc

**Figure 1:**
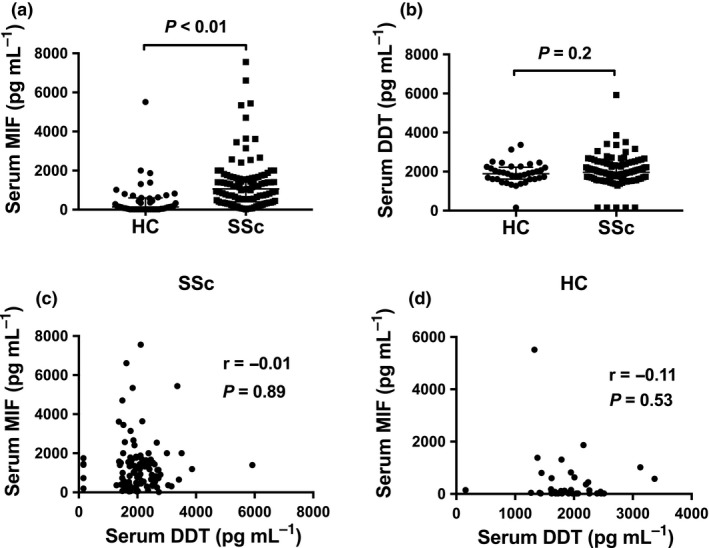
Serum MIF and DDT in SSc and HC. **(a)** Serum MIF concentrations in HC (*n* = 47) and SSc (*n* = 105) samples. **(b)** Serum DDT concentrations in HC (*n* = 37) and SSc (*n* = 102) samples. Correlation between serum MIF and DDT concentrations in **(c)** SSc patients and **(d)** HC. Panels **a** and **b**: horizontal bars indicate medians, and corresponding error bars indicate interquartile ranges; medians were compared using Wilcoxon rank‐sum test. Panels **c** and **d**: correlations were examined using Spearman's correlation test.

Serum MIF concentrations were statistically significantly higher in SSc patients than in HC, with detectable MIF in 99% (104/105) of SSc compared to 68.1% (32/47) of HC cohorts (Figure 1a and Supplementary table 2). This was confirmed in multivariable linear regression analysis adjusting for age, whereby serum MIF concentration was 6.6 times higher in SSc than in HC (ratio of GM 6.6; 95% CI 3.7, 11.9; *P* < 0.01).

Serum DDT was detectable in 95.1% (97/102) and 97.3% (36/37) of SSc patients and HC. There was no statistically significant difference in serum DDT concentrations between patients with SSc and HC (Figure 1b). MIF and DDT were not significantly correlated in serum samples from SSc patients (*r* = −0.01; *P* = 0.89) or HC (*r* = −0.11; *P* = 0.53) (Figure 1c, d).

### Serum MIF and SSc clinical parameters

We next evaluated whether there were differences in serum MIF concentrations between SSc patient subsets categorised by demographics or clinical parameters. No significant difference in serum MIF concentrations was observed between diffuse and limited disease (Supplementary table 3). Serum MIF was statistically significantly increased in SSc patients with low forced vital capacity (FVC) (Supplementary table 4). However, no statistically significant difference in serum MIF concentrations was observed according to the presence of HRCT‐confirmed interstitial lung disease (ILD), low diffusing capacity for carbon monoxide (DLCO) or low carbon monoxide transfer coefficient (KCO) (Supplementary tables 3 and 4). No significant correlation was found between serum MIF concentrations and mRSS (*r* = −0.01; *P* = 0.93). Serum MIF was significantly increased in SSc patients receiving angiotensin II receptor blockers or beta blockers, while not in those with a diagnosis of systemic hypertension (Supplementary tables 3 and 5), a finding confirmed in multivariable analysis after adjusting for the presence of systemic hypertension and low FVC (Table 2). The use of these drugs was not identified as a confounder for the association between increased serum MIF and SSc compared to SLE and HC (Table 3). Of note, serum MIF was not associated with the use of these antihypertensive drugs in SLE (data not shown). We did not observe any significant difference in serum MIF concentrations according to any clinical parameters when examining subsets of patients with limited or diffuse disease separately. No significant difference in serum MIF concentrations was observed when examining any other SSc clinical parameters (Supplementary tables 2–6).

**Table 2:** Univariable and multivariable associations of serum MIF in SSc

	Serum MIF levels (pg mL^−1^) derived from univariable linear regression analyses					Serum MIF levels (pg mL^−1^) derived from multivariable linear regression analyses				
**Exposures**			**Regression coef.**	**95% CI**	***P*** **‐value**					
Age			0.99	0.98, 1	0.22			‐	‐	‐
	**GM**	**95% CI**	**Ratio of GM**	**95% CI**	***P*** **‐value**	**GM**	**95% CI**	**Ratio of GM**	**95% CI**	***P*** **‐value**
Gender										
Males	1157	733, 1826	1.00			‐	‐	‐		
Females	751	587, 960	0.6	0.4, 1.2	0.14	‐	‐	‐	‐	‐
Ethnicity										
Other	768	407, 1450	1.00			‐	‐	‐		
Caucasians	805	649, 999	1	0.5, 2.1	0.89	‐	‐	‐	‐	‐
Low FVC										
No	749	616, 911	1.00			789	613, 1014	1.00		
Yes	1104	737, 1652	1.47	0.96, 2.27	0.08	1140	697, 1862	1.45	0.8, 2.61	0.22
Systemic hypertension										
No	748	577, 971	1.00			899	660, 1224	1.00		
Yes	1102	744, 1631	1.5	0.9, 2.3	0.09	772	425, 1404	0.86	0.39, 1.9	0.71
Angiotensin II receptor blockers										
No	712	551, 920	1.00			‐	‐	‐		
Yes	1560	1058, 2300	2.2	1.5, 3.2	<0.01	‐	‐	‐	‐	‐
Beta blockers										
No	778	606, 998	1.00			‐	‐	‐		
Yes	1745	1106, 2753	2.2	1.4, 3.5	<0.01	‐	‐	‐	‐	‐
Angiotensin II receptor blockers and/or Beta blockers										
No	692	537, 891	1.00			720	547, 948	1.00		
Yes	1635	1119, 2388	2.4	1.5, 3.8	<0.01	1770	960, 3264	2.46	1.14, 5.29	0.02*

95% CI, 95% confidence interval; FVC, forced vital capacity; GM, geometric mean; MIF, macrophage migration inhibitory factor.

* The association between angiotensin II receptor blockers and/or beta blockers with serum MIF in multivariable analysis appeared to be driven by angiotensin II receptor blockers in multivariable analysis when using angiotensin II receptor blockers and beta blockers variables separately (ratio of GM 2.03; 95% CI 0.94, 4.38; *P *=* *0.07). The use of beta blockers drug was not associated with serum MIF in multivariable analysis after adjusting with systemic hypertension, the use of angiotensin II receptor blockers drug and low FVC (ratio of GM 1.51; 95% CI 0.7, 3.23; *P *=* *0.29).

**Table 3:** Univariable and multivariable associations of serum MIF in SSc and SLE

	Serum MIF levels (pg mL^−1^) derived from univariable linear regression analyses					Serum MIF levels (pg mL^−1^) derived from multivariable linear regression analyses				
**Exposures**			**RC**	**95% CI**	***P*** **‐value**			**RC**	**95% CI**	***P*** **‐value**
Age			1.01	1, 1.02	<0.01			0.99	(0.98, 1)	0.1
	**GM**	**95% CI**	**Ratio of GM**	**95% CI**	***P*** **‐value**	**GM**	**95% CI**	**Ratio of GM**	**95% CI**	***P*** **‐value**
Disease										
HC	125	73, 214	1.00			106	62, 179	1.00		
SSc	808	638, 1024	6.5	3.5, 11.8	<0.01	889	704, 1123	8.4	4.7, 15.1	<0.01
SLE	255	200, 325	2	1.1, 3.8	0.02*	252	201, 316	2.4	1.3, 4.3	<0.01**
Angiotensin II receptor blockers										
No	387	316, 474	1.00			‐	‐	‐		
Yes	391	242, 633	1.01	0.6, 1.7	0.97	‐	‐	‐	‐	‐
Beta blockers										
No	379	323, 445	1.00			‐	‐	‐		
Yes	522	262, 1040	1.4	0.7, 2.8	0.37	‐	‐	‐	‐	‐
Angiotensin II receptor blockers and/or beta blockers										
No	379	319, 449	1.00			‐	‐	‐		
Yes	422	259, 686	1.1	0.7, 1.8	0.67	‐	‐	‐	‐	‐
Gender										
Males	473	292, 768	1.00			492	336, 720	1.00		
Females	312	257, 378	0.7	0.4, 1.1	0.09	310	271, 354	0.6	0.4, 0.9	0.02
Ethnicity										
Other	321	259, 398	1.00			‐	‐	‐		
Caucasians	334	271, 413	1.04	0.8, 1.4	0.8	‐	‐	‐	‐	‐

95% CI, 95% confidence interval; GM, geometric mean; MIF, macrophage migration inhibitory factor; RC, regression coefficient.

* Ratio GM (95% CI) SSc vs SLE: 3.17 (2.37, 4.24); *P *<* *0.01.

** Ratio GM (95% CI) SSc vs SLE: 3.53 (2.6, 4.8); *P *<* *0.01.

### Serum DDT and SSc clinical parameters

**Figure 2:**
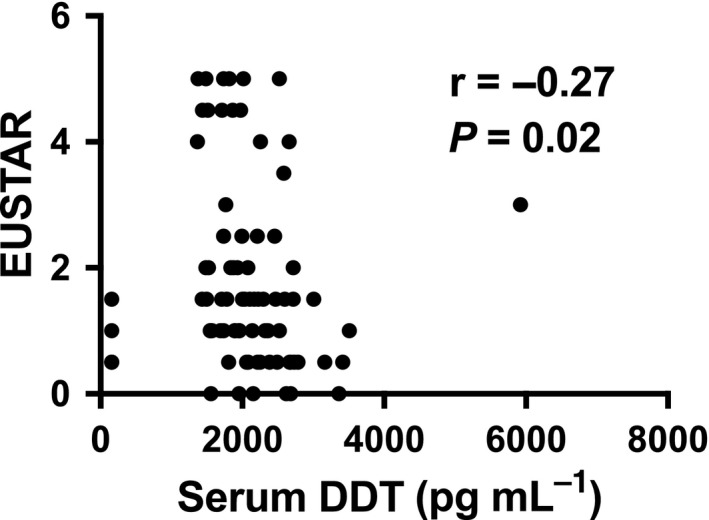
Serum DDT and SSc clinical parameters. Correlation between serum DDT concentrations and EUSTAR score in SSc. The correlation was examined using Spearman's correlation test.

We next examined serum DDT concentration according to SSc clinical parameters. No significant difference in serum DDT concentrations was observed between patients with diffuse or limited disease (Supplementary table 3), and no correlation was observed between serum DDT concentrations and mRSS score (*r* = 0.18; *P* = 0.08). We observed a statistically significant moderate negative correlation between serum DDT concentration and EUSTAR score (*r* = −0.27; *P* = 0.02; *n* = 79) (Figure 2a), which was restricted to patients with limited disease (*r* = −0.33; *P* = 0.01; *n* = 61). Serum DDT concentrations were statistically significantly higher in SSc patients with low FEV1 than in those without (Supplementary table 4). However, no significant difference in serum DDT concentrations was observed according to the presence of ILD, low FVC, low DLCO or low KCO (Supplementary tables 3 and 4). Serum DDT was also significantly lower in patients with oesophageal dysmotility (Supplementary table 2), but this is based on only four patients, so is noted with caution. We  did not observe any significant difference in serum DDT concentrations according to any other clinical parameters, including the use  of antihypertensive drugs (Supplementary tables 2–6).

### Comparison of serum MIF in SSc and SLE

**Figure 3:**
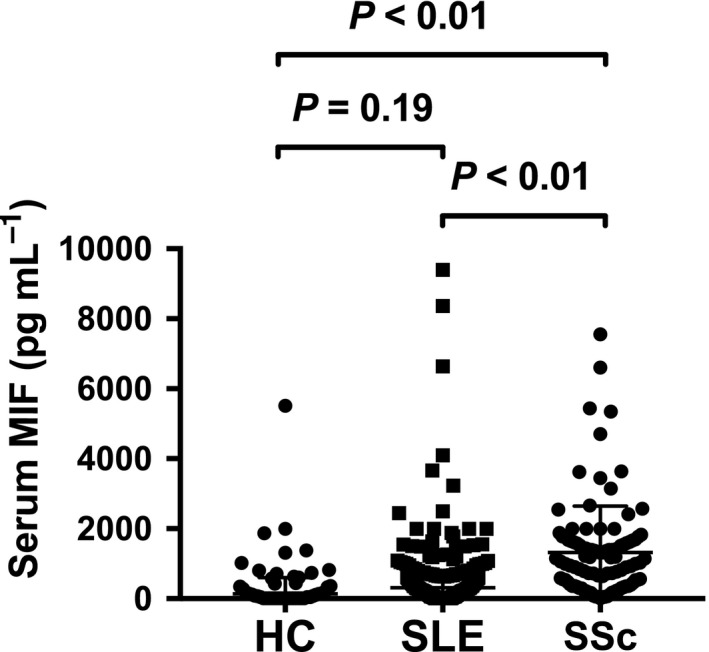
Serum MIF in SSc, SLE and HC. Serum MIF concentrations in HC (*n* = 47), SLE (*n* = 184) and SSc (*n* = 105) samples. Horizontal bars indicate medians and corresponding error bars indicate interquartile ranges; medians were compared using Dunn's multiple comparison test following Kruskal–Wallis test.

Serum MIF was significantly higher in SSc patients than in SLE patients, with detectable MIF in 99% (104/105) of SSc compared to 84.8% (156/184) of SLE patients (Figure 3a). This was confirmed in multivariable analysis adjusting for age and gender, whereby serum MIF concentrations were approximately 3.5 times higher in SSc than in SLE (Table 3).

## Discussion

Previous studies have suggested similar roles for MIF and DDT, but the extent of crossover of these two molecules in autoimmune disease is unclear. While a small number of studies have examined the potential role of MIF in the pathogenesis of SSc,^7^, ^9^, ^10^, ^13^, ^14^, ^15^ no prior published study has investigated DDT in SSc. Here, we examined concentrations of serum MIF and DDT in SSc and HC, in order to determine their clinical associations in SSc. In line with previous studies,^9^, ^15^ we found a significant increase in serum MIF concentrations in SSc patients compared to HC. However, there was no significant difference in serum DDT between SSc and HC. It is also noteworthy that serum MIF and DDT were not significantly correlated in SSc. These data suggest that MIF and DDT, while sharing similar physiological and biochemical properties,^11^ may be differentially regulated.

We also observed that increased serum MIF concentrations were associated with the use of the antihypertensive drug classes angiotensin II receptor blockers and beta blockers in SSc, and this association was independent of the presence of systemic hypertension. These findings suggest that a difference in the underlying cause of hypertension, such as increased angiotensin II levels in SSc patients, might be implicated in elevations of MIF. In this context, it is interesting to note that MIF has been shown to inhibit intracellular actions of angiotensin II in neurons.^16^ However, further studies are needed to assess the effect of angiotensin II receptor blockers and beta blocker usage on serum MIF in SSc.

We report for the first time higher serum MIF concentrations in SSc patients with low FVC, although this was not found in association with HRCT‐confirmed ILD. In SSc, FVC can be reduced because of chest wall skin thickening, independent of the presence of ILD. However, whether this is the case in our data is not established and we found no significant correlation between serum MIF concentrations and mRSS in patients with diffuse SSc. The absence of differences in serum MIF according to other clinical parameters in SSc is in line with one previous study,^14^ but conflicts with other studies which have reported higher serum MIF in patients with diffuse SSc, PAH or digital ulcers.^9^, ^10^ The small size of these vascular phenotypic subsets in our study may explain these discrepancies. Moreover, Becker *et al*., who found an association between high MIF levels and PAH, defined PAH primarily on the basis of TTE rather than RHC and therefore described a higher proportion of patients with PAH in their cohort than usually reported.^9^ The cohort examined in our study is notable for a low prevalence of PAH (4%). A larger, longitudinal study investigating the role of MIF in SSc patients with and without ILD and manifestations of obliterative vasculopathy would be of value.

We report for the first time serum DDT in SSc. We observed that serum DDT was negatively correlated with EUSTAR score, a finding restricted to patients with limited disease. Serum DDT concentrations were also significantly higher in SSc patients with low FEV1. These data are line with the above described increased serum MIF concentrations observed in SSc patients with low FVC in our study. Given the association of increased serum MIF and DDT with low FVC and low FEV1, respectively, their relationship with ILD warrants further investigation.

Our study is the first to report a comparison of serum MIF levels between two large cohorts of patients with SSc and SLE. MIF has been reported to play a role in SLE pathogenesis,^3^ as it is associated with disease activity, organ damage and glucocorticoid use.^3^, ^17^, ^18^ Our analysis revealed that serum MIF was 3.5 times higher in SSc patients than in SLE patients. This may suggest that while MIF not only can play a pathogenic role in inflammatory autoimmune diseases, such as SLE, but may also play a part in diseases that have a prominent fibrotic phase, such as that seen in SSc. This may, in turn, suggest that its biological effects may be different across autoimmune diseases with a diverse range of clinical phenotypes.

Caveats apply to the interpretation of this study. Firstly, this is a single centre study, although it is the largest study to date to analyse serum MIF in a well‐characterised SSc cohort.^9^, ^10^, ^14^ Secondly, the HC cohort was not age‐matched to the SSc cohort. However, multivariable analysis allowed adjustment for age as a potential confounding factor. Thirdly, the subset of SSc patients receiving angiotensin II receptor blockers and/or beta blockers was of modest size. Finally, the SSc cohort was characterised by a lower skin score than in previously reported studies of serum MIF in SSc,^10^, ^14^, ^15^ with only one patient having mRSS >18. This may be explained by the strong predominance of patients with limited disease (>75%) and the particularly long‐standing prevalent disease of those with diffuse disease (median [IQR] disease duration: 10.5 [5.7, 15.5] years) in our cohort. Future research examining clinical associations of serum MIF and DDT in larger cohorts of early incident diffuse SSc patients would be of value.

In conclusion, we report marked elevations of MIF in the serum of patients with SSc compared to both SLE and HC, while no significant difference in serum DDT was observed. Higher levels of MIF were associated with the use of angiotensin II blockers and beta blockers, and low FVC, but not with any other clinical parameters measured. Given the multiple reported roles for MIF in immune and autoimmune responses,^3^ this suggests that MIF is not associated with specific clinical phenotypes, but instead with the presence of SSc *per se*. This is supported by the significantly higher levels of MIF in SSc compared to SLE patients. These findings highlight the value of future investigations into how MIF and DDT may contribute to clinical and pathological outcomes in SSc, and the mechanisms through which MIF and DDT may contribute to innate immunity, autoimmunity or fibrosis in this disease.

## Methods

### Patients and clinical assessments

Adult patients attending the Monash Scleroderma Clinic between August 2015 and August 2017 fulfilling the 2013 ACR/EULAR criteria for SSc were recruited into this study. These patients were also part of the Australian Scleroderma Cohort Study. Patients were studied annually, when data on organ involvement, drug treatment, serum laboratory markers (creatinine, erythrocyte sedimentation rate (ESR) and C‐reactive protein (CRP)) and results of pulmonary function tests, high‐resolution computed tomography (HRCT) chest, transthoracic echocardiogram (TTE) and right heart catheter (RHC) were collected. Interstitial lung disease was confirmed on HRCT. PAH was confirmed on RHC as a mean pulmonary artery pressure (PAP) ≥25 mmHg at rest and a pulmonary capillary wedge pressure ≤ 15 mmHg. Pericardial effusion was diagnosed on TTE. Low FVC, low forced expiratory volume in one second (FEV1), DLCO (corrected for haemoglobin and gender) and low KCO (=DLCO/alveolar volume ratio) were all defined as <80%. At TTE, low left ventricular ejection fraction (LVEF) was defined as <55%, and abnormal systolic PAP (sPAP) was defined as >40 mmHg. Gastric antral vascular ectasia (GAVE) and reflux oesophagitis were confirmed on endoscopy. Oesophageal stricture was confirmed on either endoscopy or barium swallow. Oesophageal dysmotility was confirmed using manometry or barium swallow. Bowel dysmotility was diagnosed using barium studies or nuclear medicine studies. Scleroderma renal crisis (SRC) was defined as the presence of at least two of new‐onset systemic hypertension, rising creatinine or microangiopathic anaemia. Patients were classified as limited or diffuse SSc according to the LeRoy criteria.^19^ Extent of skin involvement was assessed using the modified (mRSS).^20^ Overall disease activity was assessed using the EUSTAR activity index, and EUSTAR score > 2.5 was considered as active disease.^21^ Screening results for anti‐centromere, anti‐topoisomerase I and anti‐RNA polymerase III antibodies were recorded at the initial study visit.

Adult patients attending the Monash Lupus Clinic (Melbourne, Australia) between June 2015 and July 2017 were recruited as an autoimmune disease control group. Patients were eligible if they fulfilled either the 1997 American College of Rheumatology (ACR) revised criteria or the Systemic Lupus International Collaborating Clinic (SLICC) criteria, and clinical data and serum samples were obtained as previously described.^22^ Healthy adult volunteers were enrolled in a HC group, gender‐ and ethnicity‐matched to the SSc cohort. Written informed consent was obtained from all participants. This study was approved by the Human Research Ethics committee of Monash Health.

### Serum cytokine quantification

Whole blood samples were collected by venepuncture, with a median [IQR] (range) time interval between clinical visit and sample collection of 0 [0, 34] (0, 364) days. Serum was isolated and stored at −80°C until further use, as previously described.^23^ Serum MIF and DDT concentrations were quantified using the human MIF DuoSet^®^ enzyme‐linked immunosorbent assay (ELISA) kit (R&D Systems; Minneapolis, MN, USA) and DDT ELISA kit (Aviva Systems Biology, San Diego, CA, USA), respectively, according to the manufacturer's protocols. Serum samples with undetectable MIF and/or DDT levels were given an arbitrary value of half the lowest standard value (15.63 and 156.25 pg mL^−1^, respectively) for statistical analysis.

### Statistical analysis

Statistical analysis was performed using Stata 14.2 (StataCorp, College Station, Texas, USA) and GraphPad (Prism V.7.0d, San Diego, CA, USA) software. Normally distributed variables were described as mean and standard deviation (SD). Non‐normally distributed variables were summarised as median with interquartile range [IQR], and Wilcoxon rank‐sum or Kruskal–Wallis (followed by Dunn's multiple comparison test) tests were used when comparing differences in continuous data between two or more than two groups, respectively. Spearman's correlation test was used to examine correlation between two continuous variables. Categorical data were described as number (frequency). Differences in proportions were compared using Pearson's chi‐squared test or Fisher's exact test where appropriate.

Linear regression analysis was used to examine associations between clinical parameters as exposure and log_10_‐transformed serum cytokine levels as outcome, as previously described.^24^ Results are presented as geometric mean (GM) and ratio of GM. GM and ratio of GM are defined as the antilog of the mean of log_10_‐transformed cytokine, and the antilog of the regression coefficient derived from linear regression analysis, respectively. Bootstrap methods with 50 repetitions were incorporated in a linear regression model to derive robust 95% confidence intervals (CI). A *P*‐value of <0.1 for association between potential confounders and both exposure and outcome variables in univariable analysis was used as a cut‐off for inclusion into a multivariable model. A *P*‐value <0.05 was considered statistically significant.

## Ethics approval and consent to participate

Written informed consent was obtained from all participants. This study was approved by the Human Research Ethics committee of Monash Health.

## Conflict of interest

The authors have no conflict of interest to declare.

## Availability of data

Reasonable requests to view the dataset used in this manuscript can be made in writing to Dr Fabien Vincent (fabien.vincent@monash.edu).

## Funding

This work was supported by a Project Grant from the National Health and Medical Research Council of Australia (grant number 1068040) and grants from the Lions Rheumatism and Arthritis Medical Research Foundation. There is no other financial support or other benefits from commercial sources for the work reported in the manuscript. The authors have no conflict of interest to declare.

## Authors’ contributions

Each individual named as an author has made substantial contributions to the conception and design of the study, acquisition of data or analysis and interpretation of data. EL, EFM, TL, JS, GSN and JH designed the experiments. FBV, JS, AH, GSN and RKR prepared patient clinical and healthy control datasets. EL, RM, TL and JH performed experiments. EL, FV, RM and RKR analysed the data. FBV, EL, TL and JH drafted the manuscript. All authors edited and approved the final version of the manuscript to be submitted.
