# Supplementary material for: Analysis of serum macrophage migration inhibitory factor and D‐dopachrome tautomerase in systemic sclerosis
Source: Clin Transl Immunology. 2018 Dec 6;7(12):e1042. doi: 10.1002/cti2.1042 (PMC6283235; doi:10.1002/cti2.1042)
Supplement: Supplementary file 1 [file CTI2-7-e1042-s001.pdf]

Supplementary Tables

Supplementary table 1. Participant demographics and disease duration in SSc, SLE and HC cohorts.

	**HC (N=47)**	**SSc (N=105)**	**SLE (N=184)**	***P*-value**
**Age**, *mean (SD)*	37.6 (10.6)	60.1 (13.9)	44.9 (14)	<0.01
**Female**	34 (72.3%)	87 (82.9%)	167 (90.8%)	<0.01
**Caucasian**	33 (70.2%)	86 (83.5%)	75 (41.9%)	<0.01
**Disease duration (years)**, *median [IQR]*	-	12.3 [6.8, 19.3]	10.2 [6, 17.2]	0.1

HC: Healthy control; SLE: Systemic lupus erythematosus; SSc: Systemic sclerosis.
P-values were derived using ANOVA (age), Pearson's chi-squared (gender and ethnicity) or Wilcoxon rank-sum test (disease duration).

Supplementary table 2. Serum MIF and DDT levels according to demographics in SSc.

	SSc patients (N=105)					
	n	Serum MIF (N=105)		n	Serum DDT (N=102)	
		Median [IQR]	*P*-value		Median [IQR]	*P*-value
Demographics						
**Age**			0.5			0.12
<60	48	1199 [532, 1652]		47	2033 [1736, 2508]	
≥60	57	905 [399, 1548]		55	1957 [1549, 2254]	
**Gender**			0.21			0.47
Male	18	1306 [553, 2000]		17	2033 [1779, 2508]	
Female	87	998 [363, 1614]		85	1957 [1638, 2383]	
**Ethnicity**			0.63			0.9
Non-Caucasian	17	1451 [355, 1585]		17	2076 [1736, 2323]	
Caucasian	86	970 [409, 1617]		83	1957 [1640, 2455]	

DDT: D-dopachrome tautomerase; MIF: macrophage migration inhibitory factor; SSc: Systemic sclerosis.
P-values were derived using Wilcoxon rank-sum test. Values are pg mL^{-1}.

Supplementary table 3. Serum MIF and DDT levels according to clinical manifestations in SSc.

	SSc patients (N=105)					
	Serum MIF (N=105)			Serum DDT (N=102)		
	**n**	**Median [IQR]**	***P*-value**	**n**	**Median [IQR]**	***P*-value**
**Disease classification**			0.91			0.15
Limited	82	1087 [409, 1617]		80	1957 [1599, 2342]	
Diffuse	23	1064 [307, 1661]		22	2044 [1779, 2719]	
**EUSTAR**			0.4			0.22
Inactive (<2.5)	60	1048 [532, 1615]		58	2079 [1736, 2524]	
Active (≥2.5)	22	1398 [553, 2000]		21	1863 [1715, 2254]	
**PAH**			0.62			0.39
Absent	101	1103 [404, 1652]		97	1957 [1640, 2383]	
Present	5	785 [566, 1322]		5	2254 [2060, 2508]	
**Pericardial effusion**			0.96			0.44
Absent	100	1048 [431, 1630]		97	1957 [1638, 2383]	
Present	5	1436 [330, 1585]		5	2380 [1779, 2524]	
**ILD**			0.94			0.68
Absent	70	1015 [528, 1614]		68	1956 [1631, 2398]	
Present	35	1187 [330, 1696]		34	1995 [1755, 2493]	
**Systemic hypertension**			0.09			0.95
Absent	68	996 [404, 1566]		66	1989 [1691, 2380]	
Present	32	1254 [641, 2478]		31	1899 [1624, 2524]	
**Digital ulcers**			0.77			0.51
Absent	86	1148 [409, 1661]		83	1995 [1573, 2455]	
Present	14	1193 [723, 1643]		14	1934 (1818, 2297]	
**GAVE**			0.16			0.23
Absent	96	1167 [404, 1669]		93	1954 [1638, 2380]	
Present	9	664 [483, 933]		9	2254 [1957, 2508]	
**Reflux oesophagitis**			0.45			0.24
Absent	46	1029 [355, 1585]		44	1912 [1537, 2261]	
Present	59	1143 [483, 1696]		58	2036 [1715, 2493]	
**Oesophageal stricture**			0.2			0.75
Absent	96	1148 [404, 1669]		93	1957 [1691, 2380]	
Present	9	730 [528, 1187]		9	1974 [1549, 2599]	
**Oesophageal dysmotility**			0.89			
Absent	100	1103 [404, 1630]		98	2000 [1707, 2455]	0.04
Present	5	998 [566, 1407]		4	1532 [836, 1725]	
**Raynaud's phenomenon**			0.61			0.28
Absent	15	1143 [334, 1436]		13	2103 [1889, 2682]	
Present	85	1187 [528, 1661]		84	1956 [1631, 2371]	
**Calcinosis**			0.82			0.89
Absent	77	1143 [452, 1614]		74	1984 [1624, 2493]	

	SSc patients (N=105)					
	Serum MIF (N=105)			Serum DDT (N=102)		
	**n**	**Median [IQR]**	***P*-value**	**n**	**Median [IQR]**	***P*-value**
Present	23	1153 [541, 1827]		23	1954 [1715, 2380]	
**Synovitis**			0.85			0.88
Absent	89	1143 [452, 1643]		86	1965 [1624, 2380]	
Present	11	1153 [528, 1753]		11	2076 [1707, 2599]	

DDT: D-dopachrome tautomerase; EUSTAR: European Scleroderma Trials and Research; GAVE: Gastric antral vascular ectasia; ILD: Interstitial lung disease; MIF: macrophage migration inhibitory factor; PAH: Pulmonary arterial hypertension; SSc: Systemic sclerosis.
P-values were derived using Wilcoxon rank-sum test. Values are pg mL^{-1}.

Supplementary table 4. Serum MIF and DDT levels according to pulmonary and cardiac function tests in SSc.

	SSc patients (N=105)					
	Serum MIF (N=105)			Serum DDT (N=102)		
	n	Median [IQR]	*P*-value	n	Median [IQR]	*P*-value
Pulmonary function tests						
**FVC (%)**			0.04			0.42
Normal (≥80%)	81	905 [399, 1548]		79	1954 [1624, 2383]	
Low (<80%)	23	1451 [1153, 2000]		22	2055 [1765, 2522]	
**FEV1 (%)**			0.58			0.04
Normal (≥80%)	76	970 [446, 1615]		74	1893 [1549, 2297]	
Low (<80%)	28	1264 [407, 1739]		27	2124 [1899, 2658]	
**DLCO (%)**[#]			0.26			0.26
Normal (80-120%)	19	1397 [820, 1696]		18	2080 [1806, 2618]	
Low (<80%)	76	1048 [381, 1630]		74	1984 [1638, 2455]	
**KCO (%)**[##]			0.89			0.69
Normal (≥80%)	18	1070 [730, 1434]		11	2076 [1806, 2225]	
Low (<80%)	80	1109 [431, 1715]		79	1957 [1638, 2455]	
Cardiac function tests						
**Six minute walk distance**			0.51			0.94
Normal (≥500m)	15	1064 [409, 1512]		15	1930 [1707, 2618]	
Low (<500m)	14	1208 [785, 1451]		13	2060 [1889, 2103]	
**LVEF (%)**			0.4			0.29
Normal (55-75%)	72	1208 [615, 1724]		70	1942 [1557, 2297]	
Low (<55%)	5	553 [334, 1359]		5	2213 [1889, 2730]	
**sPAP (mmHg)**			0.28			0.34
Normal (≤40 mmHg)	61	1359 [730, 1661]		60	1954 [1537, 2372]	
Abnormal (>40 mmHg)	16	908 [402, 1609]		15	2060 [1760, 2668]	

DDT: D-dopachrome tautomerase; DLCO: corrected diffusing capacity of the lungs for carbon monoxide; FEV1: forced expiratory volume in one second; FVC: Forced vital capacity; LVEF: Left ventricular ejection fraction; MIF: macrophage migration inhibitory factor; sPAP: Systolic pulmonary arterial pressure; SSc: Systemic sclerosis.

[#] Corrected for haemoglobin and gender.

[##] DLCO corrected for lung volume.

P-values were derived using Wilcoxon rank-sum test. Values are pg mL^{-1}.

Supplementary table 5. Serum MIF and DDT levels according to use of drugs in SSc.

	SSc patients (N=105)					
	Serum MIF (N=105)			Serum DDT (N=102)		
	n	Median [IQR]	*P*-value	n	Median [IQR]	*P*-value
**Steroids**			0.48			0.85
Absent	81	1064 [452, 1643]		79	1954 [1640, 2455]	
Present	24	992 [329, 1545]		23	2033 [1525, 2323]	
**Hydroxychloroquine**			0.43			0.4
Absent	91	1153 [399, 1661]		88	1956 [1639, 2371]	
Present	14	772 [409, 1512]		14	2068 [1691, 2618]	
**Immunosuppressants***			0.59			0.24
Absent	82	996 [452, 1617]		80	1954 [1632, 2382]	
Present	23	1311 [307, 1753]		22	2098 [1691, 2719]	
**PDE5 inhibitor**			0.79			0.32
Absent	100	1103 [383, 1652]		97	1957 [1640, 2383]	
Present	5	785 [719, 1322]		5	2254 [2060, 2584]	
**ERA**			0.95			0.81
Absent	100	1103 [383, 1652]		97	1957 [1640, 2412]	
Present	5	1031 [785, 1322]		5	2060 [1873, 2254]	
**$Ca2^+$ channel antagonist**			0.36			0.62
Absent	54	996 [452 1512]		52	1987 [1591, 2382]	
Present	51	1199 [399, 1753]		50	1965 [1691, 2508]	
**Anticoagulant**			0.97			0.11
Absent	98	1103 [399, 1617]		95	1957 [1638, 2380]	
Present	7	905 [553, 1782]		7	2524 [1760, 2719]	
**Anti-platelet agent**			0.44			0.36
Absent	86	1015 [409, 1614]		83	1974 [1707, 2469]	
Present	19	1199 [399, 1882]		19	1837 [1573, 2297]	
**ACE inhibitor**			0.94			0.16
Absent	94	1109 [399, 1617]		92	1956 [1599, 2398]	
Present	11	945 [553, 1771]		10	2102 [1930, 2682]	
**Angiotension II receptor blockers**			<0.01			0.37
Absent	88	970 [362, 1530]		85	1974 [1707, 2412]	
Present	17	1579 [905, 2544]		17	1873 [1525, 2297]	
**Beta blockers**			0.04			0.63
Absent	100	1015 [383, 1582]		97	1957 [1638, 2412]	
Present	5	1782 [1696, 1882]		5	2103 [1765, 2155]	

* includes leflunomide, methotrexate, azathioprine, mycophenylate, cyclophosphamide and calcineurin inhibitors.

DDT: D-dopachrome tautomerase; ERA: endothelin receptor antagonist; MIF: macrophage migration inhibitory factor; PDE5: phosphodiesterase 5; SSc: Systemic sclerosis.

P-values were derived using Wilcoxon rank-sum test. Values are pg mL^{-1}.

Supplementary table 6. Serum MIF and DDT levels according to laboratory markers in SSc.

	SSc patients (N=105)					
	Serum MIF (N=105)			Serum DDT (N=102)		
	n	Median [IQR]	*P*-value	n	Median [IQR]	*P*-value
**ANA anti-centromere +ve**			0.49			0.74
No	62	1193 [399, 1614]		60	1989 [1677, 2515]	
Yes	42	795 [452, 1643]		41	1957 [1691, 2297]	
**Anti-topoisomerase I Ab +ve**			0.43			0.19
No	78	939 [452, 1585]		75	2033 [1736, 2412]	
Yes	25	1395 [361, 2000]		25	1806 [1516, 2493]	
**Anti-RNA polymerase III Ab +ve**			0.85			0.36
No	93	1064 [409, 1617]		91	1957 [1691, 2383]	
Yes	9	1153 [583, 1520]		9	2167 [1818, 2524]	
**Anti-Ro Ab +ve**			0.23			0.15
No	92	970 [364, 1615]		89	1954 [1638, 2383]	
Yes	11	1359 [785, 2000]		11	2167 [1889, 2682]	
**ANCA +ve**			0.77			0.38
No	75	1143 [399, 1677]		73	1957 [1640, 2361]	
Yes	27	905 [366, 1512]		27	2060 [1638, 2668]	
**CRP**			0.62			0.49
Normal (≤5 mg/l)	66	1048 [553, 1548]		64	2000 [1665, 2462]	
High (>5 mg/l)	29	1359 [334, 1882]		28	1894 [1632, 2339]	
**ESR**			0.41			0.77
Normal (≤25 mm/h)	84	1103 [469, 1615]		81	1995 [1638, 2469]	
High (>25 mm/h)	9	1359 [905, 2000]		9	1889 [1715, 2380]	
**Serum creatinine**			0.27			0.71
Low/normal (≤110 µmol/l)	92	1148 [532, 1615]		90	1984 [1638, 2469]	
High (>110 µmol/l)	5	1782 [785, 3145]		4	1912 [1762, 2087]	

ANA: antinuclear antibodies; ANCA: anti-neutrophil cytoplasmic antibodies; CRP: C-reactive protein; DDT: D-dopachrome tautomerase; ESR: Erythrocyte sedimentation rate; MIF: macrophage migration inhibitory factor; SSc: Systemic sclerosis.
P-values were derived using Wilcoxon rank-sum test. Values are pg mL^{-1}.
